# COVID-19 in Ontario Long-term Care Facilities Project, a manually curated and validated database

**DOI:** 10.3389/fpubh.2023.1133419

**Published:** 2023-02-10

**Authors:** Mahakprit Kaur, Nicola Luigi Bragazzi, Jane Heffernan, Peter Tsasis, Jianhong Wu, Jude Dzevela Kong

**Affiliations:** ^1^Africa-Canada Artificial Intelligence and Data Innovation Consortium (ACADIC), Department of Mathematics and Statistics, York University, Toronto, ON, Canada; ^2^Faculty of Health, School of Health Policy and Management, York University, Toronto, ON, Canada

**Keywords:** COVID-19, long-term care facilities, database, Ontario—Canada, manually curated and validated database

## Background and summary

In late December 2019, a novel, emerging coronavirus, termed as “Severe Acute Respiratory Syndrome-related Coronavirus Type 2” (SARS-CoV-2) was identified as the infectious agent responsible for the generally mild, but sometimes life-threatening and even fatal “Coronavirus Disease 2019” (COVID-19).

As of December 7, 2021, COVID-19 has imposed a dramatic toll of infections (more than 265 million cases) and deaths (more than 2.5 million deaths).

Long-term care facilities, including nursing homes, residential aged care facilities, retirement homes, skilled nursing facilities and assisted living communities, among others, have represented and still represent healthcare settings particularly vulnerable to the COVID-19 spread (1). For instance, in Canada, residents living in these facilities, being elderly and particularly frail, often with many co-morbidities, have been disproportionately hit by the pandemic, contributing to approximately two thirds (67%) of the entire total toll of deaths (2).

As of December 5, 2021, 11.8% and 7.0% of COVID-19 outbreaks occurred in the Ontario region have affected long-term care facilities and retirement homes, respectively, according to Public Health Ontario (PHO).

A recently published systematic review (3) has identified an array of parameters, including bed size and location in a high SARS-CoV-2 prevalence and mortality area, and number of staff members, as variables predicting COVID-19 related outcomes.

However, in some cases, findings were contrasting, with a number of studies reporting that higher staffing was associated with a higher mortality rate and other investigations obtaining opposite results. Discrepancies in both the direction and magnitude of the effect could be found also for other parameters, such as quality indicators, like star rating, and ownership, or pandemic preparedness indicators, including implementation of public health interventions for controlling and managing prior infections and the number of previous outbreaks occurred in the facility.

Such conflicting findings may depend on the specific nature of the jurisdiction and the setting of each long-term care facility. As such, local data is of paramount importance to inform public health workers, policy- and decision-makers and relevant stakeholders in a data-driven and evidence-based fashion.

Several databases exist, mainly dedicated to (non-pharmaceutical and pharmaceutical) public health interventions (4, 5), underlying biological mechanisms, in terms of pathways and cascades (6), but, to the best of authors' knowledge, no one specifically on long-term care facilities. Specifically, there are websites that provide information for each long-term care home in Ontario such as the location of the home, type of facility, and general statistics pertaining to the care offered. However, the information is limited as the focus of this data is to provide guidance for people looking to send their loved ones to a long-term care home to assist with their daily needs. In contrast, British Columbia has one comprehensive resource curated by Seniors Advocate BC that is sponsored by the province of British Columbia called the Long-Term Care Facilities Quick Facts Directory (7). It contains detailed information regarding the facility, rooms, funding, care offered (e.g., direct care hours), licensing, incidents, resident profiles, and vaccine coverage that is specific to each long-term care home. Since this information is compiled into one reliable resource, it makes it possible for relevant information to be quickly accessed and analyzed. In Ontario, no such counterpart was found. Further, it was difficult to access relevant data that was directly available online. The only publicly available data pertaining to long-term care homes offered by the Ministry of Long-Term Care is data regarding the long-term care home location and data for publicly reported COVID-19 cases (MLTC datasets) (8). The present database was devised and implemented to fill in this gap.

## Methods

The dataset consists of 74 variables collated from over 30 sources verified by the Ontario Ministry of Health. The data was collected and compiled using a ranked source approach where original documents pertaining to each long-term care home, such as accountability agreements, were prioritized. For long-term care homes where the individual documents could not be located, sources such as The Healthline (thehealthline.ca) (9), that include annual reviews, were used. This ensured that the relevant data for each long-term care home that was available in one database but not another could be compiled into one collective dataset. The major data sources used include Long-Term Care Home Service Accountability Agreements (L-SAA) found on the LHIN websites, Ministry of Long-Term Care Inspection Reports (10), CIHI Your Health System (11), HQ Ontario Long-Term Care Performance (12), The Healthline (9), AdvantAge Ontario (13), Ontario Health Coalition (14), and Toronto Star (15). After reviewing literature to determine the relevant variables and based on available systematic reviews and published evidence (2), data regarding resident demographics, facility characteristics, region classifications, and COVID-19 cases and deaths were collected.

### Review of comparable datasets

Before beginning the collecting process, available data was reviewed and representatives of the Ministry of Long-Term Care and individual long-term care homes were contacted. The existing data publicly available online for Ontario was found to be limited in information, not as extensive as the data available in other provinces, or focusing on an overall region rather than being specific to each long-term care home.

After reaching out to the Ministry of Long-Term Care, it was found that in order to get more data, each long-term care home must be contacted. As a result, 364 homes were contacted with a response rate of 5.62% of homes that agreed to provide the necessary information for the research. Due to the low response rate, the focus of curating data shifted solely toward collecting and compiling data that was found online. It was found that different organizations, such as HQ Ontario, the Healthline, AdvantAge Ontario, and Ontario Health Coalition had collected data regarding a specific aspect of long-term care homes such as facility performance indicators, room classifications, case mix index (CMI), or bed classifications, respectively. Therefore, one of the aims of the dataset was to combine all the information into one complete dataset that can be accessed in one place.

All of the long-term care homes in Ontario are classified under the 14 Local Health Integrated Networks, or LHINs, that are responsible for overseeing the operation of the homes. After contacting the LHINs and examining their websites, it was found that they provided publicly available Long-Term Care Home Service Accountability Agreements (explained in Data Collection Process). By individually analyzing each accountability agreement for the 627 long-term care homes, it was possible to extract information such as number and type of classification beds, construction dates of the homes, and if the home was accredited. The variables are available in Schedule A of the agreement, under the heading “Description of Home and Beds.” This information was not observed to be present in currently available datasets.

The Ministry of Health and Long-Term Care maintains a website containing all the reports conducted in long-term care homes (Reports on Long-Term Care Homes) (10). The website contains all of the inspections done in the home and the inspector's reports. This resource was looked at from the perspective of aiding research pertaining to the impact of COVID-19 on long-term care home residents. As a result, information for inspections related to Sufficient Staffing, Infection Prevention and Control, and Orientation and Training was extracted and compiled. By quantifying qualitative data, it became possible to utilize the data for research that requires observing and analyzing trends. The process required reading through over 2,000 inspection reports that were written for inspections conducted in 2019 and 2020 and were classified under Complaints or Critical Incident Inspections. An extraction of data from the long-term care home reports to advance research pertaining to the COVID-19 pandemic and long-term care homes in Ontario does not seem to have been completed before.

### Data collection process

**Figure 1:**
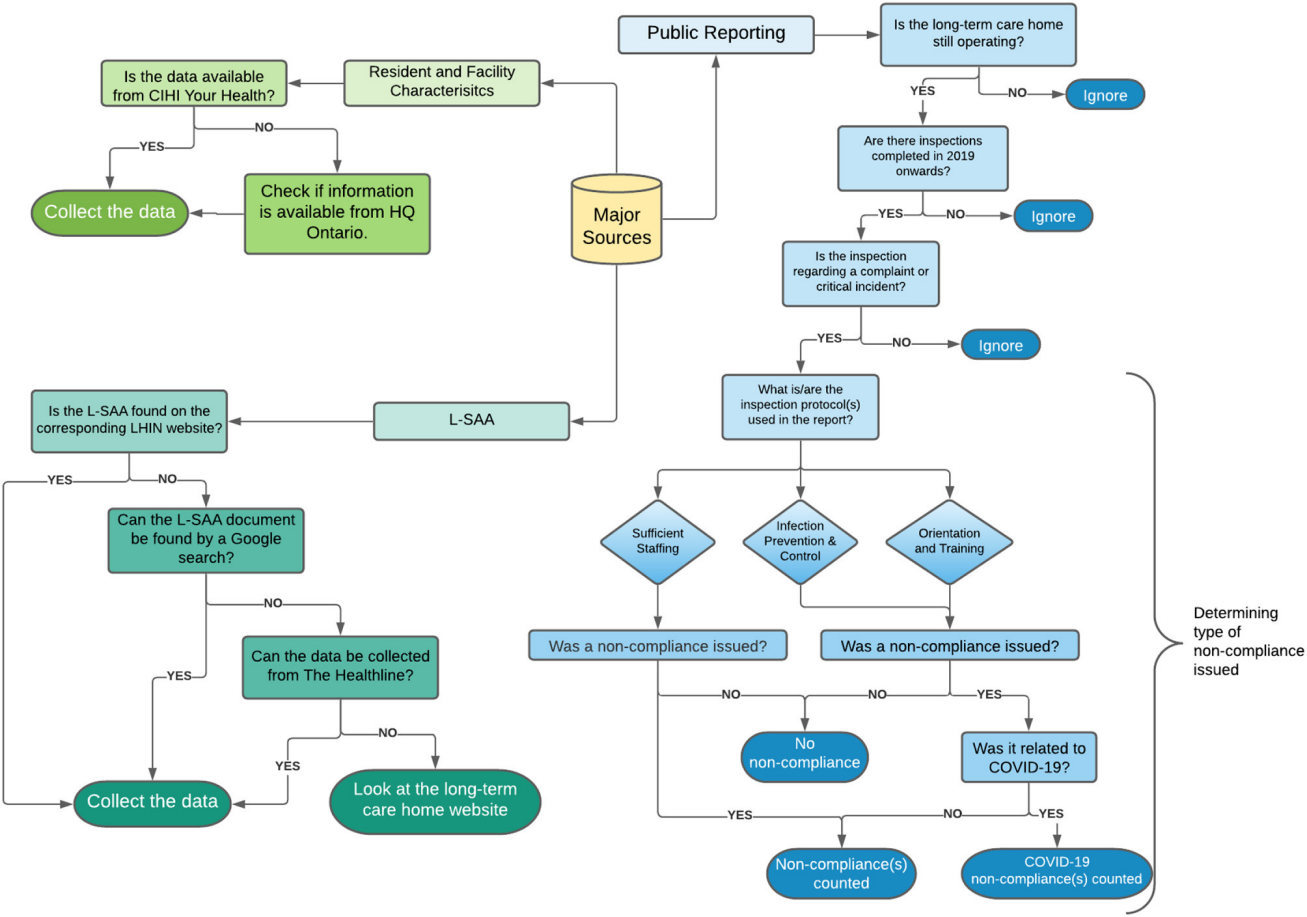
Overview of data collection process. The flow-chart pictorially represents the process by which data sources were identified, collated and compiled within a single, comprehensive manually curated and validated database. The Public Reporting steps correspond to the website titled “Reports on Long-Term Care Homes” which is maintained by the Ministry of Health and Long-Term Care. The process outlined describes the classification of each report as it pertains to sufficient staffing (SS), Infection Prevention and Control (IPAC), and Orientation and Training (OAT). Data from the Long-Term Care Home Service Accountability Agreements (L-SAA) was collected from the Local Health Integration Network (LHIN) website, by a Google search, The Healthline, or the individual website of the home. Data pertaining to resident and facility characteristics were collected through CIHI Your Health system and HQ Ontario.

The process adopted to systematically identify, collate and compile data sources is pictorially shown in Figure 1. Data collected on resident demographics, long-term care facility characteristics and quality of care indicators was compiled from CIHI Your Health System (www.yourhealthsystem.cihi.ca) (11) and HQ Ontario Long-Term Care Performance (http://www.hqontario.ca) (12). AdvantAge Ontario and Ontario Health Coalition contained datasets pertaining to Case Mix Index (CMI) and classification of the long-term care home beds, respectively. For the CIHI Your Health data, explanation of the measures can be found in their Technical Notes for Contextual Measures (PDF) (16). Most notably, for the variable “Long-Term Care Facility Location,” the designation as rural or urban is dependent on the facility's statistical area classification. Second, the facility size classification has three possibilities: small, medium, and large. Small is designated by facilities with 1–29 beds, medium as facilities with 30–99 beds and large facilities have 100 or more beds. This classification can also be adjusted and eventually be re-categorized by the researcher as the total number of beds in each long-term care home are provided in the dataset.

Long-Term Care Home Service Accountability Agreements (L-SAA) are service accountability agreements between the Local Health Integration Network (LHIN) and the Long-Term Care Home that falls within that region. It is a yearly agreement that outlines the operations of the long-term care home in order for the LHIN to continue to provide funding. Of particular interest was Schedule A of the agreement which outlined factors such as accreditation of the home, classification of licensed beds, and information regarding the home's construction. The agreement for the homes was found on their respective LHIN's website, analyzed, and the relevant information was extracted. Priority was given to the latest agreements, such as 2019–2020, to reflect and include contemporary data. Some accountability agreements were not found on the LHIN websites or were named under another long-term care home. In that case, a Google search was performed by searching the name of the long-term care home and typing “L-SAA filetype:pdf” to directly locate the agreement pdf from the internet. For the agreements that could not be located on the websites, an alternative source was used. The Healthline (thehealthline.ca) (9) provides information on local health services in Ontario, is reviewed annually, and contains service profiles created by the LHINs. Lastly, if specific information was not found through the agreements or the Healthline, then the long-term care home websites were analyzed for the data. By having a systematic approach of prioritizing agreements, then the Healthline, then the long-term care home websites, consistency and reliability of the data was ensured.

In 2018, the long-term care home sector underwent a transition from comprehensive, annual inspections to issue-specific inspections with a focus on complaints and critical incidents. Resident Quality Inspections (RQIs) are considered a comprehensive inspection of the home, and, after 2018, there were only nine conducted in Ontario (17). Of the different types of inspections, data for complaints, critical incidents, and resident quality inspections was collected. Further, the inspection reports for long-term care homes in Ontario, part of the Ministry of Long-Term Care, were screened to identify if an inspection was completed relating to “Sufficient Staffing,” “Infection Prevention and Control (IPAC),” or “Orientation and Training (OAT)” and if the inspection resulted in a non-compliance issued for the long-term care home. IPAC and OAT inspections were further divided into general non-compliance(s) and COVID-19 related non-compliance(s).

### Standards for inclusion

The sources deemed to be eligible were restricted to websites created or approved by the Ministry of Health and Long-Term Care. The aim was to collect reliable and validated data relevant to explaining the effects of COVID-19 on the number of long-term care home resident cases and deaths. As a result, some variables such as wait-times for the long-term care homes and avoidable emergency department visits, were omitted. However, since all the sources are provided within the dataset, it is possible to easily access them, saving time.

For the inspection reports, standards for inclusion consisted of keywords that determined if an inspection will count as a relevant non-compliance or not. First, all the reports were screened to determine if an inspection for “Sufficient Staffing,” “Infection Prevention and Control,” (IPAC) or “Orientation and Training” occurred. Since all inspections cite the Long-Term Care Homes Act of 2007, failure to comply by the standards falling under 2007 S.O. 2007, c.8, s. 8 (Nursing and personal support services) was recorded in order to have an objective criteria for the sufficient staffing category. For IPAC, a non-compliance was recorded if the home failed to comply with O.Reg 79/10, s. 229 of the Act. Further, it was identified as a COVID-19 related non-compliance if the report stated that the licensee failed to follow the directives, such as Directive #3, given by the government in 2020. For OAT, a non-compliance was recorded if the home failed to train their staff on infection prevention and control measures or if the licensee failed to keep their staff up-to-date with specific COVID-19 procedures. To help with the search process, relevant keywords such as “staffing mix,” “fewer than the scheduled staffing complement,” or “short-staffed” were searched for since this meant that the required number of staff, such as Personal Support Workers or Registered Nurses, were not present at all times. However, since not all reports included the keywords, all of the documents were manually screened for compliance or non-compliance to each of the policies to ensure accurate reporting. Additionally the source had inspection profiles for 653 homes. Since some of the homes were closed down or not operating in 2019 or later, they were excluded from the dataset. In the end, the data was collected for 627 homes.

## Usage notes

The COVID-19 pandemic is still ongoing and is still disproportionately affecting long-term care facilities. Within the COVID-19 in Ontario Long-term Care Facilities Project, a manually curated and validated database with over 70 relevant variables from over 30 sources was devised and implemented. This verified database is shared for any data mining effort, to test hypotheses or generate new ones about the determinants and predictors of outbreaks occurred in long-term care facilities. The structure of the database has been designed for use by biomedical, biomathematical and social scientists, to ensure broad accessibility to public health workers, decision- and policy-makers and other relevant stakeholders, (re-)use of data and high methodological transparency and reproducibility.

## Data availability statement

The original contributions presented in the study are included in the article/supplementary material, further inquiries can be directed to the corresponding author. The database can be accessed at https://tinyurl.com/27zke95t.

## Ethics statement

Ethical review and approval was not required for the study on human participants in accordance with the local legislation and institutional requirements. Written informed consent for participation was not required for this study in accordance with the national legislation and the institutional requirements.

## Author contributions

JDK designed the research. MK conducted literature search and data collection. All authors analyzed data and wrote the paper.
